# Determinants of early initiation of breastfeeding in Côte d’Ivoire: a hospital-based study

**DOI:** 10.1186/s12887-025-06190-7

**Published:** 2025-10-10

**Authors:** Kouakou Kouamé Cyprien, Djivohessoun Augustine, Djoman Isabelle, Folquet Amorissani

**Affiliations:** Department of Pediatrics, Université Félix Houphouët-Boigny, and Mother–Child Service, Cocody University Hospital, Abidjan, Côte d’Ivoire

**Keywords:** Early initiation of breastfeeding, Cesarean section, Maternal education, Neonatal outcomes, Côte d’ivoire

## Abstract

**Background:**

Early initiation of breastfeeding (EIBF), defined as putting the newborn to the breast within the first hour of life, is a cost-effective and evidence-based intervention to reduce neonatal morbidity and mortality. In Côte d’Ivoire, EIBF coverage remains suboptimal despite international recommendations.

**Methods:**

We conducted a cross-sectional analytical study from June to July 2022 at Abobo Houphouët-Boigny Regional Hospital in Abidjan. A total of 320 mother–infant dyads were consecutively enrolled. Data were collected using structured questionnaires and medical records. Associations between independent variables and EIBF were first explored using chi-square or Fisher’s exact tests. Variables with *p* < 0.20 were included in a multivariate logistic regression model. Adjusted odds ratios (aORs) with 95% confidence intervals (CIs) were reported.

**Results:**

The prevalence of EIBF was 61%. In bivariate analysis, maternal age < 20 years, maternal education, religion, unplanned pregnancy, vaginal delivery, and a 5-minute Apgar score ≥ 7 were significantly associated with EIBF. In the multivariate model, two independent predictors remained significant: higher maternal education increased the likelihood of EIBF (aOR = 2.20; 95% CI: 1.37–3.93; *p* = 0.005), whereas cesarean delivery markedly reduced it compared with vaginal delivery (aOR = 0.08; 95% CI: 0.04–0.14; *p* < 0.001).

**Conclusions:**

Although the prevalence of EIBF in this hospital was higher than the national estimate, it remains below international targets. Maternal education and mode of delivery are key determinants. Strengthening prenatal counseling and adapting post-cesarean protocols to promote immediate skin-to-skin contact are essential to increase EIBF coverage in Côte d’Ivoire.

## Introduction

Breastfeeding is universally recognized as the optimal form of infant feeding, providing essential nutrients and immune protection. Early initiation of breastfeeding (EIBF), defined as placing the newborn to the breast within the first hour after birth, is one of the most cost-effective interventions to reduce neonatal morbidity and mortality [1, 2].

According to UNICEF, only 46% of newborns worldwide are breastfed within the first hour of life, far below the recommended target of at least 70% by 2030 [3]. In West and Central Africa, prevalence is even lower (≈ 47%) [3].

Global health initiatives, including the WHO/UNICEF Global Nutrition Targets 2025 and the Sustainable Development Goals, emphasize early and exclusive breastfeeding as key strategies to improve child survival and development [1, 2]. The Baby-Friendly Hospital Initiative (BFHI) sets standards for maternity practices supporting EIBF [2]. Despite these commitments, progress remains uneven. Barriers such as cesarean delivery, inadequate counseling, and lack of continuous skin-to-skin contact continue to limit EIBF [4–6].

Evidence from sub-Saharan Africa highlights determinants of delayed initiation that include maternal sociodemographic characteristics, obstetric practices, and cultural beliefs [7–12].

In Côte d’Ivoire, national surveys indicate only 36.6% of newborns are breastfed within one hour of life [13]. Abobo Houphouët-Boigny Regional Hospital is a major referral center in Abidjan (~ 6,540 deliveries in 2021). This study aimed to identify maternal, neonatal, and contextual determinants associated with delayed initiation of breastfeeding at this hospital.

## Methods

### Study design and setting

We conducted a cross-sectional, descriptive, and analytical study over a two-month period, from June 1 to July 31, 2022, at Abobo Houphouët-Boigny Regional Hospital, a secondary-level referral facility in Abidjan, Côte d’Ivoire.

### Study population

The study population included all mother–newborn dyads admitted to the delivery ward during the study period. A dyad was defined as a mother who delivered a live infant, regardless of mode of delivery (vaginal or cesarean), gestational age (term or preterm), or complications, provided the interview could be conducted within 72 h after birth.

### Sample size calculation

The minimum sample size was estimated using Cochran’s formula for a single population proportion: *n* = Z² × p × (1 − p)/d², with Z = 1.96 (95% confidence), *p* = 36.6% (national estimate for EIBF in Côte d’Ivoire), and precision d = 0.055. Applying these parameters yielded a sample size of ≈ 295 dyads. Allowing for 10% non-response, the target sample was 325 dyads. Ultimately, 320 dyads were consecutively enrolled.

### Eligibility criteria

Inclusion: all live mother–infant dyads with verbal informed consent. Exclusion: dyads where the newborn died before data collection or was transferred before the interview.

### Data collection and variables

The dependent variable was EIBF (breastfeeding within one hour after delivery, per WHO). Independent variables covered three domains: maternal sociodemographic characteristics (age, marital status, education level, occupation); obstetric factors (gravidity, parity, gestational age, number of antenatal care visits, prenatal decision to breastfeed, prior breastfeeding experience, counseling received, partner support, pregnancy pathologies, mode of delivery); and neonatal factors (sex, birth weight, 5-minute Apgar score, need for resuscitation). Postnatal practices recorded included skin-to-skin contact (delivery room, operating theater, recovery), rooming-in, immediate post-cesarean practices (routine separation vs. not), and clinical reasons preventing EIBF. Data were collected using a standardized questionnaire administered in French by trained interviewers within 72 h after delivery, and cross-checked with medical records.

### Statistical analysis

Data were entered and analyzed using Epi Info^®^ version 7.2.5 (CDC, Atlanta, USA). Categorical variables were described as frequencies and percentages; continuous variables as means with standard deviations. Associations between EIBF and independent variables were first examined using chi-square or Fisher’s exact tests. Variables with *p* < 0.20 in bivariate analysis were included in a multivariable logistic regression model (enter method). Crude and adjusted odds ratios (ORs and aORs) with 95% confidence intervals (CIs) were reported; *p* < 0.05 was considered statistically significant. Missing data were rare (< 5%) and handled by case-wise deletion. Given the cross-sectional design, associations cannot be interpreted as causal; residual confounding may persist.

### Ethical considerations

The study complied with the Declaration of Helsinki. Verbal informed consent was obtained from all participating mothers, confidentiality was maintained, and no additional procedures were performed beyond routine care. We acknowledge that the protocol was not submitted for prior approval to an institutional research ethics committee.

## Results

### Characteristics of the study population

A total of 320 mother–infant dyads were included. The mean maternal age was 26.6 ± 5.1 years, with most mothers aged 20–34 years (45.9%). Nearly 37% were younger than 20 years, while 17% were aged ≥ 35 years. The majority lived in free union (87%), with only 13% legally married. More than half (55%) had no formal education; 31% had secondary and 7% higher education. Half were self-employed, 35% housewives, and a minority held salaried positions or were students. Muslims predominated (59.4%) compared with Christians (40.6%) (Table 1).

**Table 1:** Maternal sociodemographic characteristics (*N* = 320)

Variables/Categories	*n*	%
Maternal age (years)		
< 20	118	36.9
20–34	147	45.9
≥ 35	55	17.2
Marital status		
Married	279	87.0
Free union	4	1.3
Other (single/divorced/widowed)	37	11.7
Education level		
None	176	55.0
Primary	22	6.9
Secondary	100	31.3
Higher	22	6.9
Occupation		
Self-employed	159	49.7
Housewife	112	35.0
Salaried	22	6.9
Unemployed	18	5.6
Student	9	2.8
Religion		
Christian	130	40.6
Muslim	190	59.4

*Abbreviations:* *N* total sample size; *n* number; % percentage

On the obstetric side, 37.5% were multigravidas, 32.8% paucigravidas, and 29.7% primigravidas. The most common parity was multiparity (49%), followed by nulliparity (34.4%). Most deliveries were at term (96%), and 94.4% had fewer than eight antenatal care (ANC) visits. A prenatal decision to breastfeed was reported by 73.4% of mothers; 65% had prior breastfeeding experience. Only 36.6% reported receiving specific counseling on breastfeeding; 18.8% declared their pregnancy had been planned. Partner support was reported by 82.5%. Hypertension and HIV infection occurred in 0.9% and 1.9% of pregnancies, respectively. Vaginal delivery predominated (71.6%) (Table 2).

**Table 2:** Obstetric characteristics (*N* = 320)

Variables/Categories	*n*	%
Gravidity		
Primigravida	95	29.7
Paucigravida	105	32.8
Multigravida	120	37.5
Parity		
Nulliparous	110	34.4
Pauciparous	53	16.6
Multiparous	157	49.0
Gestational age (weeks)		
< 37	12	4.0
≥ 37	308	96.0
Antenatal care visits		
< 8	302	94.4
≥ 8	18	5.6
Prenatal decision to breastfeed		
Yes	235	73.4
No	85	26.6
Previous breastfeeding experience		
Yes	208	65.0
No	112	35.0
Received counseling on breastfeeding		
Yes	117	36.6
No	203	63.4
Planned pregnancy		
Yes	60	18.8
No	260	81.2
Partner support		
Yes	264	82.5
No	56	17.5
Pregnancy pathologies		
Hypertension	3	0.9
HIV infection	6	1.9
Mode of delivery		
Vaginal	229	71.6
Cesarean	91	28.4

Regarding neonatal characteristics, the sex ratio was balanced (51.2% boys vs. 48.8% girls). Most newborns had a birth weight ≥ 2500 g (90%). The 5-minute Apgar score was ≥ 7 in 95% of cases. Neonatal resuscitation was required in only 1.2% of deliveries (Table 3).

**Table 3:** Neonatal characteristics (*N* = 320)

Variables/Categories	*n*	%
Sex		
Male	164	51.2
Female	156	48.8
Birth weight		
< 2500 g	32	10.0
≥ 2500 g	288	90.0
5-minute Apgar score		
< 7	16	5.0
≥ 7	304	95.0
Neonatal resuscitation		
Yes	4	1.2
No	316	98.8

### Prevalence of early initiation of breastfeeding

Overall, 61% of newborns were breastfed within the first hour after birth. Among those who did not benefit from EIBF, some received age-appropriate formula before the first breastfeed, illustrating partial adherence to recommendations.

### Bivariate analysis

In bivariate analysis, several maternal, obstetric, and neonatal factors were significantly associated with EIBF. Mothers younger than 20 years were more likely to initiate breastfeeding within the first hour compared with those aged 20–34 years (67.8% vs. 55.8%; OR = 1.69, 95% CI: 1.02–2.81). Compared with secondary education, EIBF was less frequent among mothers with only primary education (31.8% vs. 58.3%; OR = 0.34, 95% CI: 0.13–0.90), while those with higher education tended to initiate earlier. Muslim mothers were more likely to practice EIBF than Christian mothers (65.8% vs. 53.8%; OR = 1.65, 95% CI: 1.04–2.60). EIBF was also more frequent when the pregnancy was unplanned (63.8% vs. 48.3%; OR = 1.89, 95% CI: 1.07–3.32). Mode of delivery was a major predictor: vaginal delivery strongly favored EIBF compared with cesarean delivery (76.0% vs. 23.1%; OR = 0.09, 95% CI: 0.05–0.17). Newborns with a 5-minute Apgar score < 7 were much less likely to be breastfed within the hour compared to those with Apgar ≥ 7 (18.8% vs. 63.2%; OR = 0.13, 95% CI: 0.04–0.48). Parity, number of ANC visits, prior breastfeeding experience, and birth weight were not significantly associated with EIBF (Table 4).

**Table 4:** Bivariate analysis of factors associated with early initiation of breastfeeding (≤ 1 h)

Variable/Categories	≤ 1 h *n* (%)	> 1 h *n* (%)	Crude OR (95% CI)	*p*-value
Maternal age				
< 20 years	80 (67.8)	38 (32.2)	1.69 (1.02–2.81)	0.137
20–34 years (Ref.)	82 (55.8)	65 (44.2)	1.0	–
≥ 35 years	33 (61.1)	21 (38.9)	1.26 (0.67–2.39)	
Maternal education				
None	113 (64.2)	63 (35.8)	1.30 (0.79–2.15)	
Primary	7 (31.8)	15 (68.2)	0.34 (0.13–0.90)	0.010
Secondary (Ref.)	49 (58.3)	35 (41.7)	1.0	–
Higher	17 (77.3)	5 (22.7)	2.46 (0.84–7.20)	
Religion				
Christian (Ref.)	70 (53.8)	60 (46.2)	1.0	–
Muslim	125 (65.8)	65 (34.2)	1.65 (1.04–2.60)	0.042
Planned pregnancy				
Yes (Ref.)	29 (48.3)	31 (51.7)	1.0	–
No	166 (63.8)	94 (36.2)	1.89 (1.07–3.32)	0.038
Mode of delivery				
Vaginal (Ref.)	174 (76.0)	55 (24.0)	1.0	–
Cesarean	21 (23.1)	70 (76.9)	0.09 (0.05–0.17)	< 0.001
5-minute Apgar score				
≥ 7 (Ref.)	192 (63.2)	112 (36.8)	1.0	–
< 7	3 (18.8)	13 (81.2)	0.13 (0.04–0.48)	0.001

*OR* crude odds ratio, *CI *confidence interval, *Ref*. reference category

*p*-values from chi-square or Fisher’s exact tests as appropriate

### Multivariable analysis

In the multivariable model, two factors remained significantly associated with EIBF: higher maternal education (aOR = 2.20; 95% CI: 1.37–3.93; *p* = 0.005) and cesarean delivery (aOR = 0.08; 95% CI: 0.04–0.14; *p* < 0.001) (Table 5).

**Table 5:** Multivariable analysis of factors associated with early initiation of breastfeeding (≤ 1 h)

Variable/Categories	aOR (95% CI)	*p*-value
Maternal education		
Secondary (Ref.)	1.0	–
Higher	2.20 (1.37–3.93)	0.005
Mode of delivery		
Vaginal (Ref.)	1.0	–
Cesarean	0.08 (0.04–0.14)	< 0.001

## Discussion

This study conducted at Abobo Houphouët-Boigny Regional Hospital shows that just over six in ten newborns were breastfed within the first hour after birth (61%). This prevalence is higher than the national estimate (36.6%) [13] but remains below operational targets for maximizing neonatal survival [1–3, 8]. In the multivariable model, higher maternal education was positively associated with EIBF, while cesarean delivery was strongly unfavorable.

These findings align with international literature confirming the negative effect of cesarean delivery on breastfeeding initiation and continuation [4–6]. Rates observed in other African contexts vary—comparable to Ghana, Nigeria, and Senegal [7, 12, 14], yet slightly lower than in Rwanda and Ethiopia [8, 9]—likely reflecting differences in delivery protocols, staff training, and BFHI integration [2, 11].

The favorable association between higher maternal education and EIBF echoes studies in Africa and Asia linking education to improved breastfeeding knowledge, attitudes, and practices [8–10, 15], although paradoxical patterns have been reported in some West African settings [11, 12]. This “paradox” may reflect sociocultural norms favoring breastfeeding in less educated populations, reduced exposure to breast-milk substitute marketing, or greater medicalization of childbirth among more educated women, leading to iatrogenic delays in skin-to-skin contact [16]. In our hospital setting, the overall favorable effect of education suggests that health education received during ANC and comprehension of public health messages play a key role.

Mechanistically, vaginal delivery facilitates immediate mother–infant proximity and continuous skin-to-skin contact, promoting early breastfeeding, whereas cesarean section introduces delays linked to operating and recovery room processes, postoperative monitoring, and pain [5, 6].

Neonatal vitality at 5 min (Apgar ≥ 7) was strongly associated with EIBF in bivariate analysis, consistent with East African observations [17, 18]. Implications include: (i) systematic skin-to-skin in operating and recovery rooms (including after spinal anesthesia) through coordinated obstetric–anesthesia–neonatal care; (ii) reinforced antenatal messages about breastfeeding within the first hour, tailored to education level and leveraging community health workers; and (iii) explicit integration of EIBF into neonatal resuscitation pathways (early colostrum once stabilized), consistent with WHO/EENC and BFHI frameworks.

## Conclusion

At this referral center in Abidjan, 61% of newborns were breastfed within the first hour—above the national estimate but below public health targets. After adjustment, higher maternal education facilitated EIBF while cesarean delivery markedly hindered it. Optimization of post-cesarean pathways (immediate skin-to-skin, effective analgesia, rooming-in, early colostrum) and strengthened prenatal counseling should be prioritized to increase EIBF coverage and reduce neonatal morbidity and mortality in Côte d’Ivoire.

## Data Availability

The datasets used and/or analyzed during the current study are available from the corresponding author upon reasonable request.

## Abbreviations

EIBF: Early initiation of breastfeeding
OR: Odds ratio
aOR: Adjusted odds ratio
CI: Confidence interval
ANC: Antenatal care
BFHI: Baby-Friendly Hospital Initiative
